# Rates of the Trans-radial Approach in Elective and Emergency Coronary Angiography in Iraq: A Cross-Sectional Study

**DOI:** 10.7759/cureus.41193

**Published:** 2023-06-30

**Authors:** Ameen M Mohammad, Nazar A Shammo, Saad Y Saeed

**Affiliations:** 1 Department of Internal Medicine, University of Duhok, Duhok, IRQ; 2 Department of Internal Medicine, Azadi Cardiac Center, Duhok, IRQ; 3 Department of Community Medicine, University of Duhok, Duhok, IRQ

**Keywords:** iraq, middle east, trans-femoral approach, trans-radial approach, coronary angiography

## Abstract

Background and aims

The trans-radial access is becoming the default approach in many cardiac centers worldwide. Data from the Middle East, including Iraq, on the trends and rates of the use of trans-radial access are scarce. The aim of this study is to determine the rates of trans-radial approach (TRA) versus transfemoral approach (TFA) in patients with coronary artery syndromes undergoing coronary angiography and/or percutaneous coronary intervention (PCI) in Iraq.

Methods

In this multicenter prospective study, we collected 885 cases of coronary artery disease undergoing coronary angiography/PCI from three main cities of the Kurdistan Region in Iraq from 2022 to 2023.

Results

Of the total sample, 57.2% were diagnostic coronary angiography and 42.8% were PCI, 57.1% of all cases were TFA and 42.9% were TRA, and 64.3% of PCI cases were performed through TFA. Eghty-two percent of total emergency PCI included (primary PCI) cases underwent the procedure through the TFA, and only 18% of such cases were through the TRA. The overall crossover rate between both approaches happened in 14 (3.6%) cases.

Conclusions

Despite its main benefits, the radial access use in the Cath lab is yet underused in our region. Further steps in training programs are indicated to popularize the use of radial access among interventional cardiologists in addition to transfemoral access.

## Introduction

Coronary artery disease has had high morbidity and mortality for a long time. To date, coronary angiography and percutaneous coronary intervention (PCI) are the golden standard diagnostic and therapeutic strategies for coronary artery disease, respectively [1-3].

The ideal site for coronary angiography and PCI is a topic of discussion and in-depth research. They have typically been performed via a transfemoral route, but trans-radial access is gradually becoming more common in practice [4], in part because it carries a reduced risk of bleeding and vascular problems than the transfemoral approach (TFA), particularly in patients with acute coronary syndromes (ACS). Additionally, it is linked to bettering ACS-related outcomes, raising the standard of medical care, and cutting costs [5-8]. Due to its larger size and the recent use of larger diagnostic and angioplasty guiding catheters, the femoral artery has traditionally been the preferred site of approach. The range of interventions has been greatly expanded by the use of improved coronary hardware and the creation of novel anticoagulants [9].

Trans-radial access (TRA) has recently been recommended by the European Society of Cardiology recommendations as the usual technique for all PCI, regardless of clinical presentation (recommendation: Class I, level A) [10]. Although TRA can significantly reduce access site complications, it has some drawbacks, including smaller vessel size, vessel spasm, radial artery occlusion, and more techniques for guiding catheter placement, which might lengthen the procedure and exposes the patients and staff to more radiation [11]. Due to the paucity of TRA data from the Middle East region, this study is conducted to determine the rates and trends of TRA in the Kurdistan Region of Iraq.

## Materials and methods

Patients and methods

We collected in this cross-sectional multicenter study in Iraq 885 patients from Duhok, Erbil, and Sulaymaniyah cardiac centers between 2022 and 2023. After obtaining relevant clinical data, angiographic and procedural characteristics, we divided the patients based on the aim of the study into two groups for comparison, transfemoral and trans-radial diagnostic coronary angiography and therapeutic coronary interventions. The study included patients taken for both emergency and elective diagnostic and therapeutic coronary angiographies. Exclusion criteria were cases with coronary artery bypass graft cases, co-existing peripheral angiography, negative Allen test, and non-palpable radial arteries.

The right radial and right femoral access points were our favorites in this study. The insertion of sheathes was done based on standard protocols. In most cases, six French sheathes and catheters were used in both diagnostics and therapeutics angiographies. The period of time from the point of sterilization to procedure completion was referred to as the procedure time. From the moment a patient was admitted to the cardiac unit until they were discharged, their hospital stay was calculated.

Following the procedure, the Terumo radial compression device was used to apply pressure to the area for two hours while the radial sheath was removed in the cardiac units. After removing the femoral sheath for 15 to 20 minutes and then bandaging the area for four hours, a qualified medical assistant applied manual compression to achieve hemostasis during the femoral operation. In the absence of eventful complications, the timetable hospital stay protocol in the centers was four to six hours for diagnostics, six to eight hours for elective PCIs, and 24 hours for emergency PCIs. Devices for vascular closure in TFA were not employed due to unavailability. Patients were returned to the ward, where bleeding and other problems at the radial site and femoral site were carefully monitored. Patients for whom we were unable to complete the procedure through TRA for any reason had crossed over to a femoral approach, and the groin was kept ready.

Statistical analysis

SPSS Statistics version 20.0 (IBM Corp. Released 2011. IBM SPSS Statistics for Windows, Version 20.0. Armonk, NY: IBM Corp.) was used to analyze the data after they had initially been entered into a Microsoft Excel spreadsheet (Microsoft, Washington, USA). The data were described using frequency tables, range, mean, and standard deviation (SD). The association between categorical data was examined using the Chi-square test, and the mean differences were examined using the unpaired t-test. Statistics were considered significant for p-values under 0.05.

Ethical approval

The Iraqi Kurdistan Higher Council of Medical Specialties research ethics committee gave the study its seal of approval (approval number: 208). A written informed consent was given by every patient enrolled in the trial.

## Results

A total of 885 patients were enrolled. The age ranged from 28 to 90 years (mean of 59.1 ± 9.3), and 61.2% were males and 38.8% were females. The BMI ranged from 17 to 35 (mean of 25.8 ± 2.4). We performed 506 (57.2%) diagnostic angiographies and 379 (42.8%) PCI. Of total procedures, the TRA were 380 (42.9%) and 505 (57.1%) TFA. Only 135 (35.6%) of the total (379) PCIs were primary PCIs for STEMI cases. Demographic and procedural characteristics of all patients are represented in Table 1.

**Table 1:** Baseline demographic characteristics and procedure related of all patients IHD: ischemic heart disease, PCI: percutaneous coronary intervention, BMI: body mass index

Characteristics	N (%)
Age, mean, years	59.1 ± 9.3
Male	542 (61.2)
Female	343 (38.8)
BMI, mean	25.8 ± 2.4
Hypertension	454 (51.3)
Diabetes mellitus	295 (33.3)
Smoking	264 (29.8)
IHD	131 (14.8)
Heart failure	63 (7.1)
Previous angiography	314 (35.5)
Renal impaired	43 (4.9)
Stroke	20 (2.3)
Diagnostic angiography	506 (57.2)
PCI	379 (42.8)
Femoral approach	505 (57.1)
Radial approach	380 (42.9)
Elective procedure	750 (84.7)
Emergency procedure	135 (15.3)
Local complications:	
Hematoma	17 (1.9)
Pain	111 (12.5)
Vasovagal	8 (0.9)
Times of radial:	
First time	56 (15.3)
Second or more	309 (84.7)
Crossover to femoral	8 (2.1)
Fluoroscope time (mean) in minutes	7.83 ± 7.92
Procedure time (mean) in minutes	20.6 ± 10.3
Contrast volume (mean) in ml	88.3 ± 52.0
Hospital stays (mean) in hours	6.6 ± 4.4

The comparison of patients' basic characteristics by access approach is summarized in Table 2. There were statistically significant differences in those variables (gender, BMI, hypertension, heart failure, renal impaired, and stroke) between the two approaches (p-value <0.05).

**Table 2:** Comparison of patients' basic characteristics by access approach *Based on chi-square Note: percentages are vertical, and comparisons are to be made horizontally BMI: body mass index, IHD: ischemic heart disease

Variables		Femoral Approach No. %		Radial Approach No. %		p-value*
Age in years	28-45	37	7.3	21	5.5	0.39
	46-60	271	53.7	192	50.5	
	61-75	181	35.8	151	39.7	
	76-90	16	3.2	16	4.2	
Gender	Male	289	57.2	253	66.6	0.005
	Female	216	42.8	127	33.4	
BMI (kg/m2)	<25	133	26.4	122	31.9	0.016
	25-<30	342	68.2	251	66	
	≥30	28	5.4	9	2.1	
Hypertension	Yes	244	48.3	210	55.3	0.041
	No	261	51.7	170	44.7	
Diabetes mellitus	Yes	161	31.9	134	35.3	0.291
	No	344	68.1	246	64.7	
Smoking	Yes	157	31.1	107	28.2	0.345
	No	348	68.9	273	71.8	
IHD	Yes	81	16	50	13.2	0.232
	No	424	84	330	86.8	
Heart failure	Yes	52	10.3	11	2.9	<0.001
	No	453	89.7	369	97.1	
Previous angiography	Yes	179	35.4	135	35.5	0.98
	No	326	64.6	245	64.5	
Renal impairment	Yes	13	2.6	30	7.9	<0.001
	No	492	97.4	350	92.1	
Stroke	Yes	18	3.6	2	0.5	0.003
	No	487	96.4	378	99.5	
Total		505	100	380	100	

A comparison of patients’ procedural characteristics by the access approach is shown in Table 3. There were statistical differences in p-value <0.05 in the type of procedure, case situation, and local access complications. Nearly, the diagnostic angiographic approach was similar in both approaches (TFA 51.5% and TRA 48.5%). Most of the TRA was for diagnostic (64.5%). The majority of emergency (primary) PCI was through TFA 111 (82.2%) with a p-value of <0.001.

**Table 3:** Comparison of patients’ clinical characteristics and procedure related by access approach *Based on chi-square test **Based on unpaired t-test PCI: percutaneous coronary intervention

Variables		Femoral Approach No. %		Radial Approach No. %		p-value*
Type of procedure	Angiography	261	51.7	245	64.5	<0.001
	PCI	244	48.3	135	35.5	
Case situation	Elective	394	78	356	93.7	<0.001
	Emergency	111	22	24	6.3	
Local complications	Hematoma	15	3	2	0.5	<0.001
	Pain	23	4.6	88	23.2	
	Vasovagal	2	0.4	6	1.6	
	None	465	92.1	284	74.7	
Crossover rate	Yes	6	1.2	8	2.1	0.279
	No	499	98.8	372	97.9	
		Mean	SD	Mean	SD	p-value**
Fluoroscope time, minutes		8.43	8.24	7.03	7.41	0.004
Procedure time, minutes		20.6	11	20.7	9.3	0.808
Contrast volume, ml		92.3	49.3	82.9	54.9	0.008
Hospital stays, hours		8.9	4.9	4.1	1.4	<0.001

Local hematoma occurred in 3% of TFA and 0.5% of TRA, respectively, with a p-value of <0.001. Local access pain was frequent in TRA 23.2% vs. 4.6% in TFA with a p-value of <0.001. Vasovagal is more frequent in radial access at 1.6%. The overall crossover rate between TRA and TFA happened in 14 (3.6%) cases. Eight patients in the TRA required a crossover to TFA because of severe subclavian artery tortuosity, radial loop, and inability to engage coronary ostia. Six TFA required a crossover to TRA due to severe iliofemoral tortuosity and previous femoral hematoma. The mean fluoroscopy times were 8.42 ± 8.24 and 7.01 ± 7.41 minutes in TFA and TRA, respectively, with a p-value of 0.004. The hospital stay in TFA was 8.9 hours as the mean vs. 4.1 hours in TRA with a p-value of <0.001.

## Discussion

In this study, we had primary PCI for coronary artery syndromes as well as elective and emergency coronary angiographies and angioplasties. TFA was 505 patients overall (57.1%), while TRA was 380 individuals (42.9%). Prior to 2008, the TFA was regarded as the primary artery through which cardiac catheterizations were performed. However, due to the high risk of major and minor vascular complications associated with transfemoral cardiac catheterization, trans-radial cardiac catheterization is currently on the rise worldwide [12]. At the moment, Asia and Europe are more popular in trans-radial cardiac catheterization. By Campeau, the TRA for coronary angiography was initially documented in 1989, and then TRA coronary stenting was performed by Kiemeneji in 1993 [9,13,14].

Up to the moment, according to our findings, the TRA in the country was used mainly for diagnostic angiographies and the majority of PCI approaches were TFA. In regards to emergency and primary PCI, the TFA was the predominant one (22%) vs. (6.3%) TRA. This indicates that the TRA is quite underused despite its popularity and advantages worldwide, particularly in ACS/STEMI substrates of patients. Today, TRA is preferred over TFA in STEMI according to international cardiology society guidelines [15,16]. TRA has been slowly adopted in the United States for patients with STEMI [11,17,18]. Most recently, the SAFARI-STEMI trial (Safety and Efficacy of Femoral Access Versus Radial Access in ST-Elevation Myocardial Infarction) showed no major differences in major bleeding or 30-day all-cause mortality in a population of STEMI patients who underwent primary PCI through either access [19,20]. In addition, the RIFLE-STEACS trial showed that radial access in patients with STEMI was associated with important treatment advantages, including reduced morbidity and cardiac mortality. The trial recommended TRA for these patients, provided that there is sufficient operator and center experience [21]. TRA seems to be advantageous in experienced centers. Therefore, TRA may not be superior to TFA in centers that have insufficient experience.

Based on patients' characteristics, TRA was performed more commonly in obese, males, and heart failure patients in the current study. Obese patients have a higher risk of developing local complications and bleeding in TFA and the hemostasis is challenging. Females are traditionally more prone to develop vasospasm of the radial artery. In heart failure patients, the TRA is helpful in terms of shortening the supine positions required in TFA for site hemostasis [12,22].

In terms of complications, the TFA group in this study experienced more procedure-related problems than the TRA group. Hematomas happened in 3% of TFA versus 0.5% in the TRA. In Bhat et al.'s study, hematomas were discovered in 14.5% of TFA compared to none in the TRA [22]. Similar findings were made by Choussat et al. [23]. We found also that the TRA had a higher prevalence of pain (23.2%) compared to the TFA (4.6%). This was comparable to Vefali et al.'s findings [24]. In eight (2.1%) cases, a crossover from TRA to TFA occurred due to expected causes including access failure, hostile subclavian anatomy, and poor ostial engagement. This was 4% in Kassman et al. [25]. In general, access failure was more frequent with TRA according to Kiemeneij et al. [14]. Access failure in general is usually related to radial artery puncture failure, radial artery spasm, radial loop, tortuosity of the innominate trunk, and failure to contact the coronary ostia [14]. Updated research from the American national cardiovascular data registry also demonstrated that TFA PCI had a higher rate of procedural success [26]. Both radial and femoral methods were proven to be safe and efficacious for PCI in the RIVAL study [27].

In the current study, the TFA had a longer mean fluoroscopy time than the TRA. Besides, both approaches took about the same amount of time to complete the procedure. This is opposed to Bhat et al.'s findings where the fluoroscope time and overall procedure time were significantly longer in TRA [19]. Hou et al. found no significant variations in the length of the fluoroscope or the surgical process between both approaches [28]. The increased number of PCIs performed by TFA in the current study might be the factor behind the longer fluoroscopic time that was observed.

The study's average hospital stay/duration following angiographies/angioplasties was 8.9±4.9 hours in the TFA against 4.1±1.4 hours in the TRA. This is consistent with Vefali et al. [24] and Bhat et al. [22]. Additionally, comparable to the results of a recent report from the national cardiovascular data registry in the United States [26]. However, Choussat et al. [23] found no statistically significant difference between TFA and TRA in terms of hospital stay following the procedure. In terms of the amount of contrast used during the procedures, we utilized more contrast material during TFA compared to TRA. Again, this comes in line with that of Hirzallah et al. [29] and in contrast to Hibbert et al. [30].

Limitations

Although the study is multicenter and showed important data related to rates and trends of TRA in the region, it has several limitations. Firstly, the unavailability of essential materials and equipment inside the Cath lab might influence the operator's decision and procedural endpoints. Secondly, the differences in the skills and the preferences of the operators could result in some selection biases like the use of TRA in simpler coronary lesions and fit patients. Finally, lack of post-interventional follow-up of cases for rates of radial artery occlusions and late adverse cardiac events. These limitations might be considered for future projects in the region.

## Conclusions

To conclude, the TRA is feasible in our area. It has been used primarily for diagnostic angiographies and non-emergency PCIs. It has advantages in terms of vascular complications and hospital stays. However, still it is underused in the Cath lab, particularly in emergency coronary procedures by our cardiologists. Further steps in training programs are indicated to popularize the use of TRA in addition to TFA among interventional cardiologists.
